# Improved image quality and greater diagnostic suitability in myocardial delayed enhancement CT with deep learning image reconstruction

**DOI:** 10.1007/s11604-026-01958-y

**Published:** 2026-02-17

**Authors:** Akio Yamazaki, Yasutaka Ichikawa, Satoshi Nakamura, Takanori Kokawa, Masafumi Takafuji, Mana Deguchi, Florian Michallek, Masaki Ishida, Kakuya Kitagawa, Hajime Sakuma

**Affiliations:** Department of Radiology, Mie University Hospital, 2-174 Edobashi, Tsu, Mie 514-8507 Japan

**Keywords:** Deep learning–based image reconstruction, Computed tomography, Myocardial delayed enhancement, Image quality, Hybrid iterative reconstruction, Image reconstruction

## Abstract

**Purpose:**

Myocardial delayed enhancement computed tomography (MDE-CT) is an emerging imaging modality for assessing myocardial fibrosis. However, its diagnostic performance is often limited by low contrast resolution and high image noise. Deep learning–based image reconstruction (DLIR) has recently been introduced as a novel method to enhance CT image quality. This study aimed to evaluate whether DLIR improves image quality and diagnostic suitability in MDE-CT, compared to conventional hybrid iterative reconstruction (HIR).

**Materials and methods:**

A total of 108 patients with visually confirmed myocardial delayed enhancement on CT were included. CT images were reconstructed using both HIR and DLIR. Quantitative image quality metrics included image noise, contrast-to-noise ratio (CNR), and signal-to-noise ratio (SNR). Qualitative image quality was independently assessed by two radiologists using a 5-point Likert scale (1 = poor, 5 = excellent), with scores ≥ 3 considered diagnostically suitable.

**Results:**

DLIR significantly reduced image noise (median 7.1 Hounsfield unit [HU] vs. 9.2 HU) and improved both CNR (median 3.2 vs. 2.6) and SNR (median 11.7 vs. 9.0) compared to HIR (all *p* < 0.0001). DLIR increased CNR and SNR by 26.9% and 27.1%, respectively. Qualitative scores were also significantly higher for DLIR (Observer 1: 4.2 ± 0.8 vs. 3.4 ± 0.8; Observer 2: 3.6 ± 0.8 vs. 3.2 ± 0.9; all *p* < 0.0001). The proportion of diagnostically suitable images significantly increased in both readers (Observer 1: 88.9% [96/108] to 97.2% [105/108]; Observer 2: 82.4% [89/108] to 90.7% [98/108]; both *p* < 0.03).

**Conclusion:**

DLIR significantly improves both quantitative and qualitative image quality in MDE-CT, resulting in a higher proportion of diagnostically suitable images. These improvements support the incorporation of DLIR into routine MDE-CT protocols as a robust alternative to conventional iterative reconstruction.

## Introduction

Myocardial delayed enhancement (MDE) imaging plays a key role in identifying myocardial tissue alterations, such as fibrosis and infarction [1–3]. While late gadolinium enhancement cardiac magnetic resonance imaging (LGE-CMR) is the established reference standard for this purpose, recent advances in cardiac computed tomography (CT) have indicated that MDE-CT may server as a feasible alternative modality, particularly for patients with contraindications to CMR [4–6]. Despite these developments, a key limitation of MDE-CT lies in its suboptimal image quality relative to LGE-CMR, primarily due to the inherently lower iodine contrast-to-noise ratio (CNR) and reduced contrast resolution [7].

Recent improvements in CT technology, including novel image acquisition and reconstruction algorithms, have contributed to reduced image noise and improved overall image quality. Among these, deep learning–based image reconstruction (DLIR) has gained increasing attention as a next-generation technique that utilizes convolutional neural networks trained on high-quality image datasets to achieve superior noise suppression while preserving fine anatomical detail [8, 9]. Previous studies have demonstrated that DLIR outperforms conventional hybrid iterative reconstruction (HIR) in a variety of clinical CT applications, such as thoracic, abdominal, and vascular imaging, especially under low-dose conditions, where HIR often struggles to balance effective noise reduction with texture fidelity [10–13]. However, the potential of DLIR in MDE-CT remains insufficiently explored. Accordingly, this study aimed to assess whether DLIR can improve image quality and increase the proportion of images considered suitable for diagnostic interpretation in MDE-CT, compared to conventional HIR.

## Materials and methods

### Subjects

Between November 2021 and March 2023, a total of 354 patients who met the following inclusion criteria were retrospectively identified: (1) age ≥ 18 years at the time of imaging; and (2) underwent clinically indicated cardiac CT including MDE-CT, with complete reconstructed image datasets available for both HIR and DLIR. Among these 354 patients, those who did not exhibit MDE on CT as determined by review of both HIR and DLIR reconstructed images were excluded. The presence or absence of MDE was determined by a board-certified radiologist (S.N. with 12 years expertise in cardiac CT). To ensure the reliability of MDE identification, the initial determination of MDE presence was independently reviewed by a second board-certified radiologist (T.K., with 8 years of expertise in cardiac CT). All findings of MDE were confirmed without discrepancy. Subsequently, these two radiologists classified the enhancement pattern (ischemic or non-ischemic) in consensus. Next, additional exclusion criteria were applied as follows: (1) presence of implanted devices (e.g., pacemaker or implantable cardioverter-defibrillator) that significantly interfered with image interpretation and (2) reduced contrast medium administration due to impaired renal function or other clinical considerations. This retrospective study was approved by the institutional review board (approval number: H2019-207), and the requirement for written informed consent was waived due to the use of existing clinical imaging data. No patients opted out of the study.

### CT image acquisition and reconstruction

**Fig. 1:**
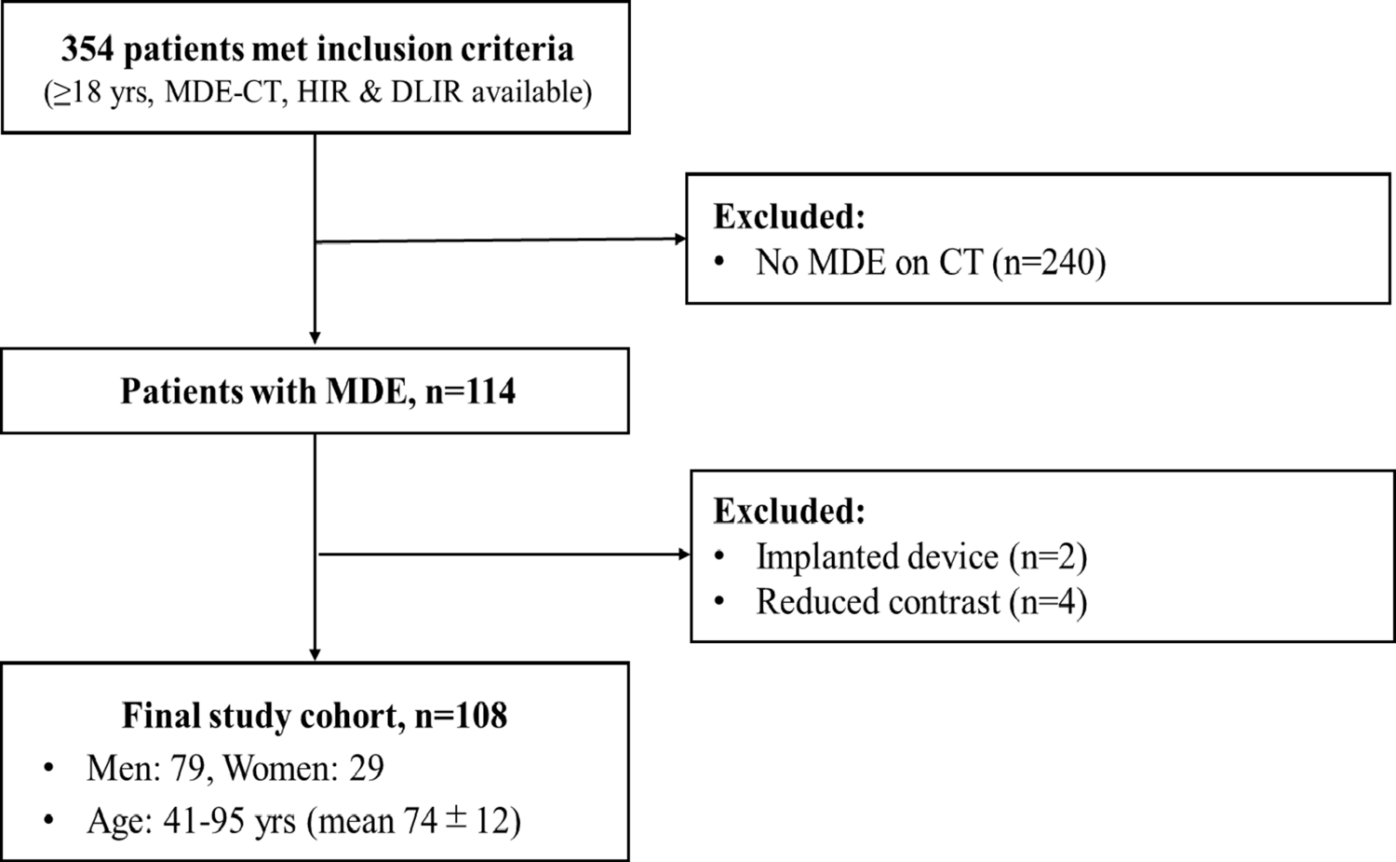
Flow diagram of patient selection. Abbreviations: MDE; myocardial delayed enhancement, CT; computed tomography, HIR; hybrid iterative reconstruction, DLIR; deep learning-based imagereconstruction

All cardiac CT examinations were performed using a 256-slice CT scanner (Revolution CT, GE Healthcare). At our institution, MDE-CT is routinely performed immediately after coronary CT angiography as part of the standard cardiac CT protocol. MDE-CT was performed 5 min after coronary CT angiography. For the coronary CT, iodinated contrast material (Iopamiron 370, 100 mL; Bayer Schering Pharma) was administered at 26 mg iodine/kg/s for 12 s. To prevent exceeding a maximum injection rate of 5.0 mL/s, the rate was capped at this value in patients weighing over 72 kg, without extending the injection duration. No additional contrast was given for the subsequent MDE-CT scan. As a result, the total iodine dose was 312 mg iodine/kg for patients ≤ 72 kg, and fixed at 22.5 g iodine for those > 72 kg. MDE-CT images were acquired using an axial, prospective electrocardiogram-triggered protocol with image acquisition targeted at 250 ms after the R-wave. Four-phase images were acquired during a single breath-hold. These images were subsequently processed using a commercially-available software (*syngo*.via VB80F, Siemens Healthcare). Non-rigid registration was first applied to correct inter-phase misalignment, followed by four-dimensional noise reduction to enhance image quality. This acquisition and post-processing technique has been previously described as an effective method for improving image quality in delayed enhancement CT [5, 14]. The scan parameters for MDE-CT were as follows: matrix, 512 × 512; tube voltage, 80kVp; tube current, 500–700 mA; gantry rotation time, 0.28 s; and *z*-axis coverage, 100–160 mm depending on the size of patient’s heart. Image reconstruction was performed using both hybrid iterative reconstruction (ASiR-V; GE Healthcare) with a 50% blending factor and a standard kernel, and deep learning image reconstruction (DLIR; TrueFidelity, GE Healthcare). TrueFidelity offers three levels of noise reduction strength (low, medium, and high); in this study, the highest level was used. For the evaluation of delayed enhancement, transaxial and left ventricular short-axis images were reconstructed with a slice thickness of 5 mm (Fig. 1).

## Quantitative image quality

**Fig. 2:**
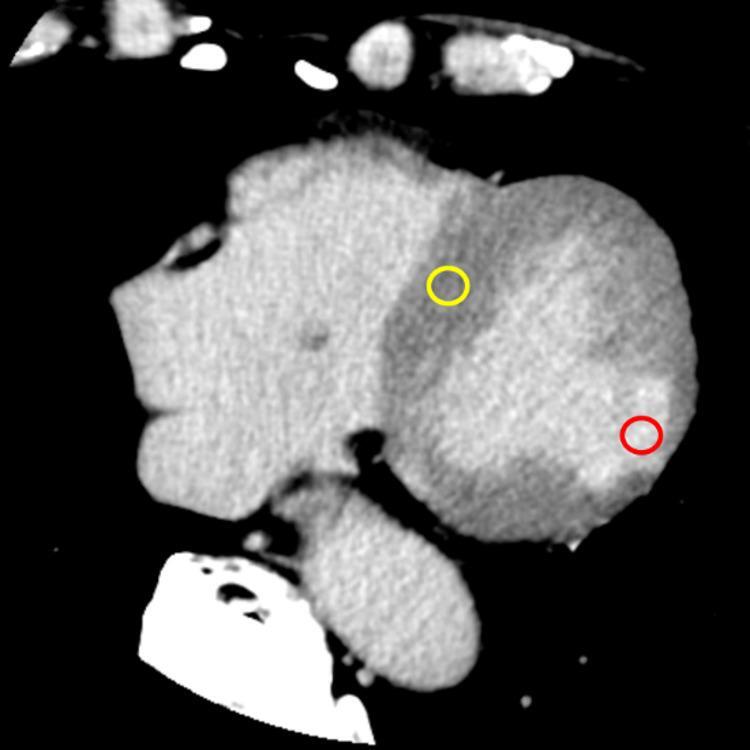
Representative axial image from myocardial delayed enhancement CT showing placement of circular regions of interest (ROIs) for quantitative analysis. The yellow circle indicates an ROI placed in the remote myocardium, while the red circle marks an ROI placed within the hyperenhanced myocardium

Quantitative analysis of image noise and tissue contrast was then performed by an experienced radiographer (A.Y., with 12 years of experience in cardiovascular CT), using a PACS viewer (PSP; PSP Corporation, Tokyo, Japan). All quantitative measurements were conducted on transaxial images reconstructed with a slice thickness of 5 mm. Circular regions of interest (ROIs) were manually placed in the following locations: (1) the remote (normal) myocardium, and (2) the hyperenhanced myocardium. For the remote myocardium, ROIs were typically placed in the ventricular septum at the mid-ventricular level on axial images, in homogeneous regions carefully avoiding artifacts, beam-hardening, and partial volume effects. The ROI size for the remote myocardium was standardized with a diameter of approximately 1 cm to ensure consistency across patients. For the hyperenhanced myocardium, the axial slice with the largest extent of delayed enhancement, irrespective of whether the pattern was ischemic or non-ischemic, was selected. On this slice, a circular ROI was manually drawn as large as possible within the hyperenhanced region, while carefully avoiding the endocardial and epicardial borders to minimize partial volume effects. A schematic example of ROI placement for quantitative measurements is shown in Fig. 2. The signal-to-noise ratio (SNR) and contrast-to-noise ratio (CNR) were calculated as:

SNR = mean of the remote myocardium / SD of the remote myocardium

*CNR = (mean of the hyperenhanced myocardium – mean of the remote myocardium) / SD of the remote myocardium.*

where SD indicates the standard deviation within the ROI. The SD measurements were used as the image noise.

## Qualitative image quality

**Fig. 3:**
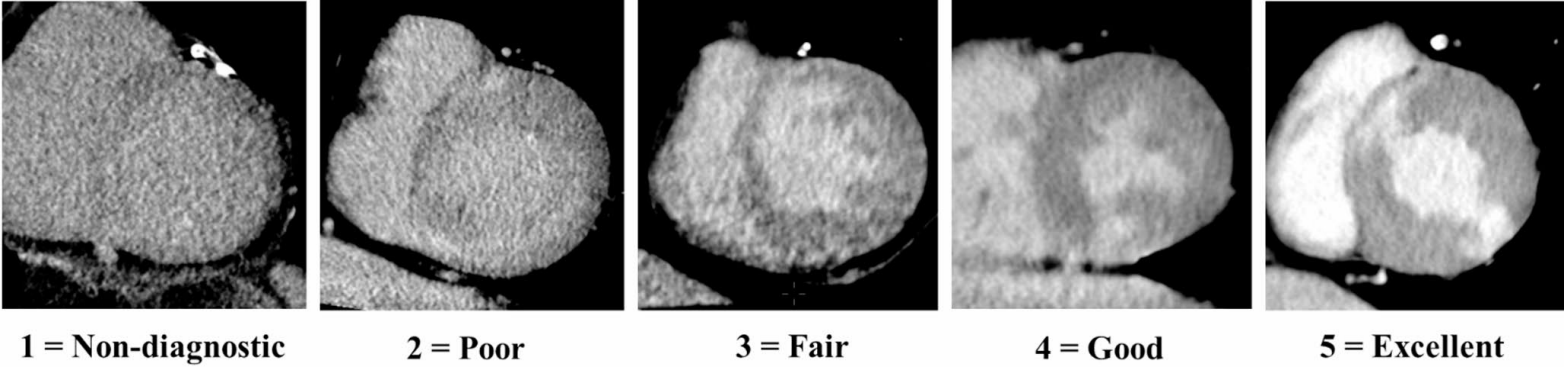
Representative short-axis myocardial delayed enhancement CT images illustrating each score on the 5-point image quality scale

Qualitative evaluation of image quality was independently performed by two board-certified radiologists (Y.I. and M.T. with 20 and 10 years of experience in cardiac imaging, respectively). Both observers were blinded to the reconstruction method (HIR or DLIR) and to each other’s evaluations to minimize potential bias. The reconstructed image datasets, comprising both transaxial and left ventricular short-axis planes of MDE-CT, were anonymized and presented in random order for each patient using a dedicated PACS viewer (PSP; PSP Corporation, Tokyo, Japan), which allowed full access to standard viewing tools. Each observer was allowed to freely adjust the window level and width to their preferred settings in each case, in order to simulate routine clinical reading conditions. Image series were evaluated in both axial and short-axis views. Overall image quality was scored using a 5-point Likert scale as follows: score 1 = non-diagnostic due to severe noise or artifacts, score 2 = poor image quality with significant limitations in interpretation, score 3 = fair, interpretable but with moderate degradation in quality, score 4 = good quality with minimal noise or artifacts, and score 5 = excellent image quality with clear depiction of myocardial structures and minimal noise. Scores of 3 or above were considered suitable for diagnostic interpretation in this study. In addition, to evaluate whether body habitus influenced image quality assessment, patients were stratified into two groups based on the median body weight, and image quality scores were compared between HIR and DLIR within each subgroup. Representative images corresponding to each score on the 5-point scale are shown in Fig. 3.

### Radiation dose

To assess radiation exposure for MDE-CT and coronary CT angiography, CT dose index (CTDIvol) and the dose length product (DLP) was recorded for each patient.

### Statistical analysis

Continuous variables are presented as mean ± SD if normally distributed or, otherwise, as median and interquartile range (IQR). The Wilcoxon matched-pairs signed-rank test was used to compare image noise, CNR, and SNR between HIR and DLIR images. The difference in the proportion of diagnostically suitable images (score ≥ 3) between HIR and DLIR was assessed using the McNemar test. A *p*-value < 0.05 was considered statistically significant. Statistical analysis was performed with MedCalc version 23.2.1 (MedCalc Software, Ltd.) and R software, version 4.5.0 (R Foundation for Statistical Computing).

## Results

### Study population

**Fig. 4:**
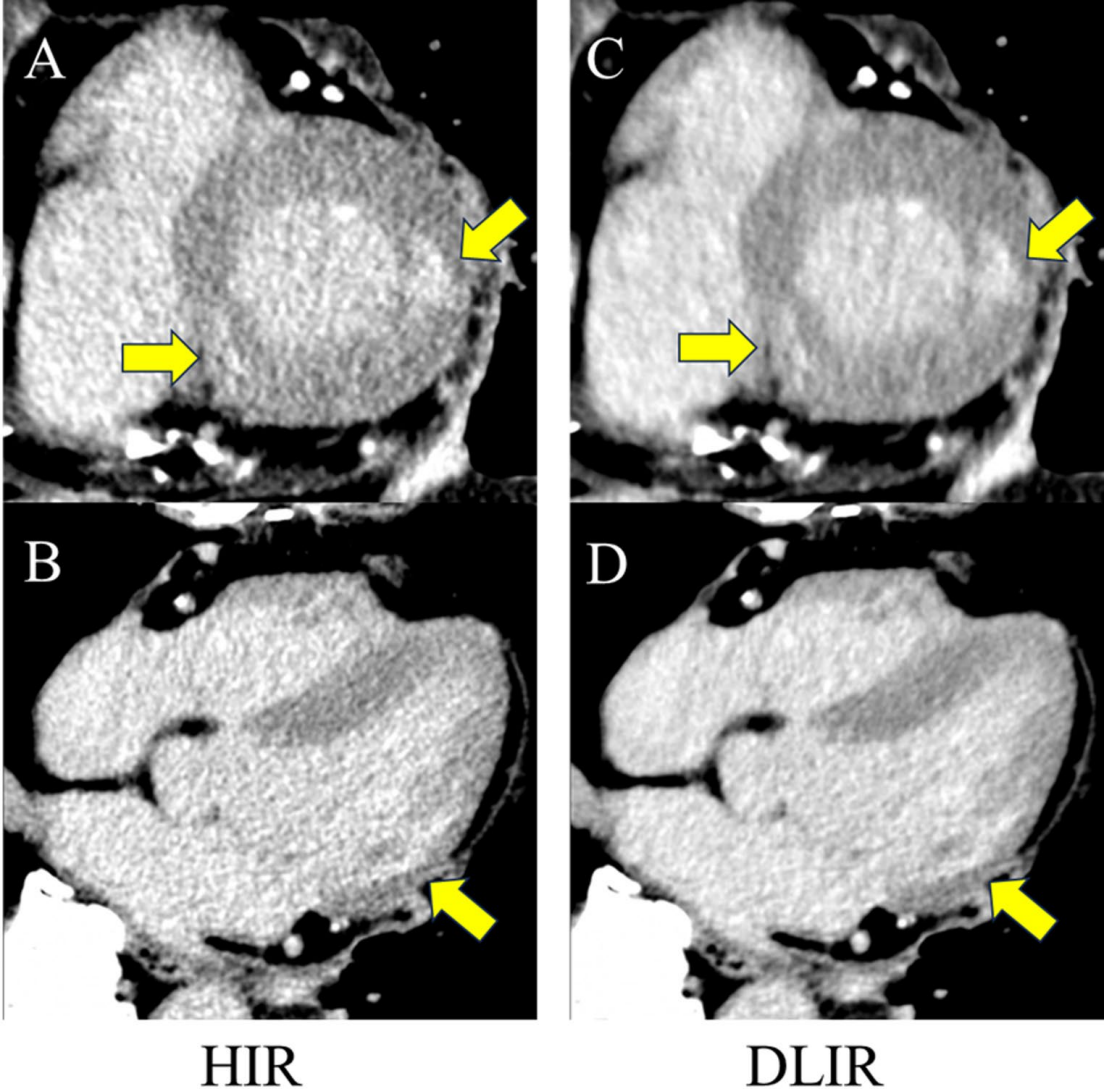
Representative delayed enhancement CT images (5-mm slice thickness). **A**　Short-axis image reconstructed with conventional hybrid iterative reconstruction (HIR); **B** transaxial image with HIR; **C** short-axis image with deep learning–based image reconstruction (DLIR); **D** transaxial image with DLIR. Compared with conventional HIR images, DLIR images demonstrate visibly reduced image noise and clearer delineation of the delayed enhancement areas in the lateral wall and posterior septum (arrows). In this representative case, overall image quality was scored as 4 for HIR and 5 for DLIR by observer 1, and 3 for HIR and 4 for DLIR by observer 2, indicating improved image quality with DLIR

The final study cohort consisted of 108 patients (79 men and 29 women), with an age range of 41–95 years (mean age, 74 ± 12 years). A detailed flowchart of patient selection is provided in Fig. 1. The patient characteristics are summarized in Table 1. To illustrate the typical appearance in image quality differences between HIR and DLIR, a representative case of myocardial delayed enhancement is presented in Fig. 4. Ischemic enhancement was observed in 86 patients (79.6%), whereas non-ischemic enhancement was identified in 22 patients (20.4%).

**Table 1:** Patient characteristics

No. of patients		108
Women / Men		29 / 79
Age (years)		72.7 ± 11.5
Body mass index (kg/m^2^)		23.1 ± 3.5
Body weight (kg)		60.5 ± 12.1
Mean heart rate (beats/min)		63.6 ± 11.0
*Risk Factor*, *n* (%)		
Hypertension		84 (77.8%)
Dyslipidemia		65 (60.2%)
Diabetes		57 (52.8%)
Current smoker		14 (13.0%)
Family history of CAD		20 (18.5%)
*Underlying conditions, n (%)*		
Ischemic heart disease		60 (55.6%)
Non-ischemic heart disease		48 (44.4%)
Valvular disease		25 (23.1%)
Aortic disease (aneurysm or dissection)		11 (10.2%)
Atrial fibrillation		8 (7.4%)
Cardiomyopathy		2 (1.9%)
Infective endocarditis		1 (0.9%)
Heart failure of undetermined etiology		1 (0.9%)

Continuous variables are expressed as the mean ± standard deviation

### Quantitative assessment

**Fig. 5:**
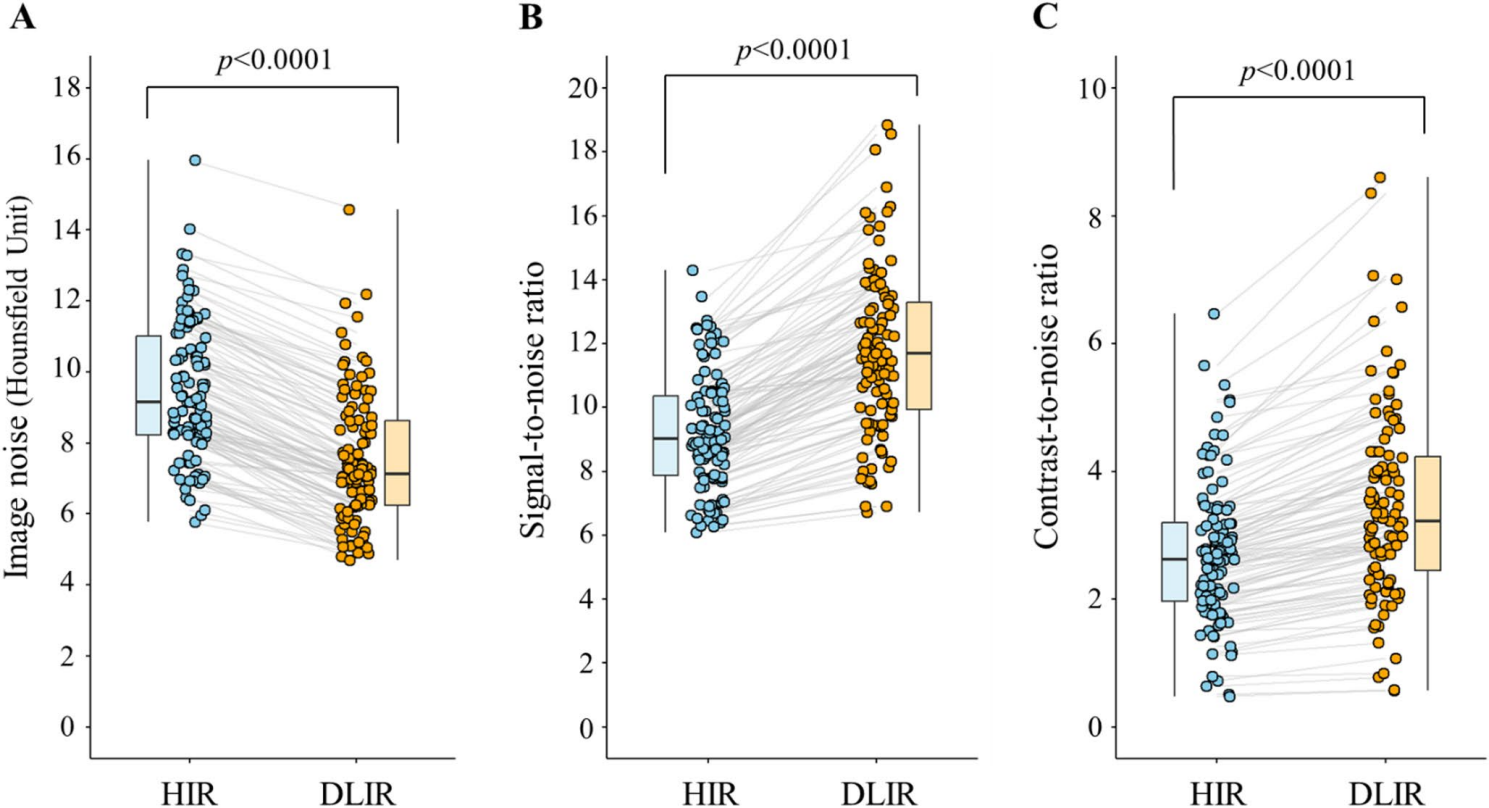
Comparison of image nose (**A**), signal-to-noise ratio (**B**), and contrast-to-noise ratio (**C**) of myocardial delayed enhancement CT images reconstructed by hybrid iterative reconstruction (HIR) and deep learning image-based image reconstruction (DLIR). Each plot displays paired data for individual patients. Gray lines connect data from the same patient. Box plots show the distribution of each parameter, with the boxes representing the interquartile range (IQR), the horizontal line within each box indicating the median, and the whiskers extending to the minimum and maximum values

Quantitative results for image noise, SNR and CNR are summarized in Fig. 5. Image noise of DLIR was significantly lower (median: 7.1 Hounsfield units [HU], IQR: 6.2–8.6 HU) compared to HIR (median: 9.2 HU, IQR: 8.2–11.0 HU), *p* < 0.0001. The mean reduction rate in image noise with DLIR compared to HIR was 20.7 ± 7.2%. The CNR of the hyperenhanced myocardium was significantly improved with DLIR (median: 3.2, IQR: 2.4–4.2) compared to HIR (median: 2.6, IQR: 1.9–3.2) (*p* < 0.0001). The SNR was also significantly higher with DLIR (median: 11.7, IQR: 9.9–13.3) than with HIR (median: 9.0, IQR: 7.8–10.4) (*p* < 0.0001). Compared to HIR, DLIR increased the CNR and SNR by 26.9 ± 12.2% and 27.1 ± 11.6%, respectively.

### Qualitative image assessment

**Fig. 6:**
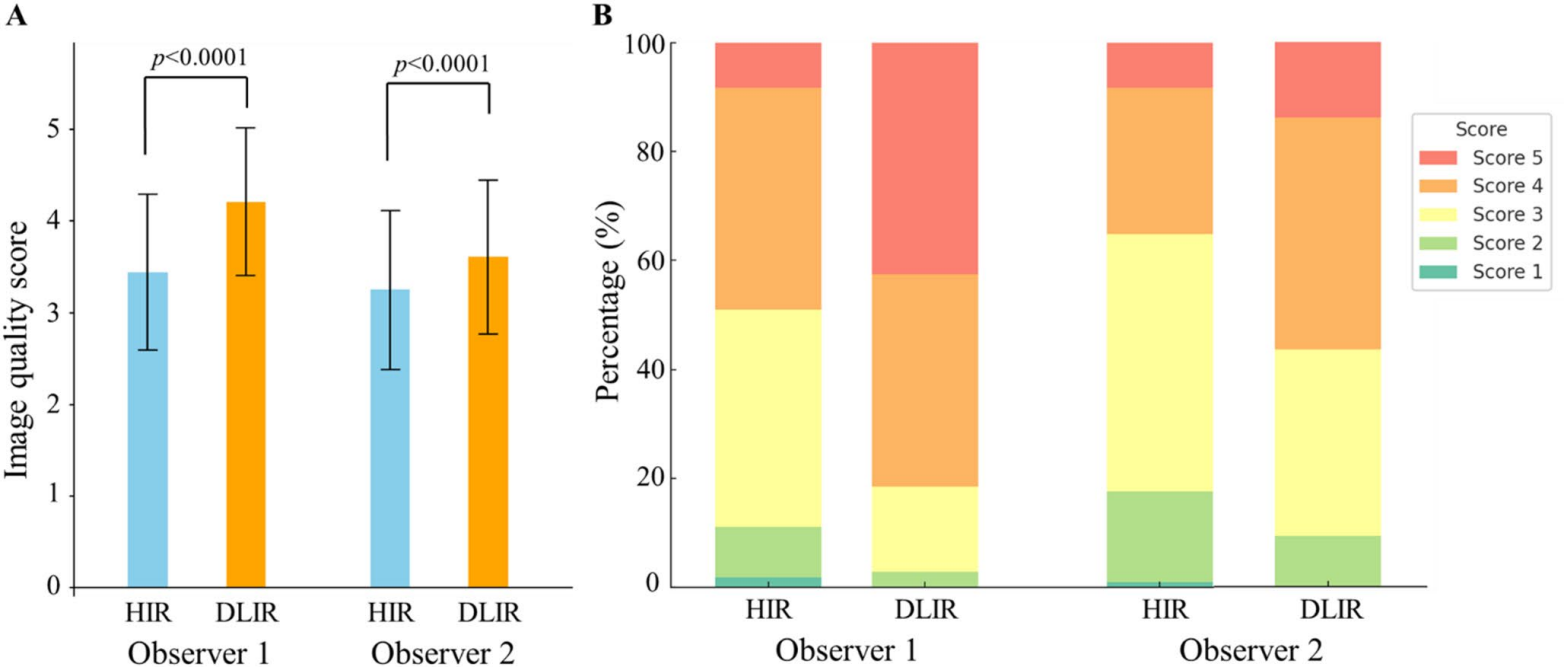
Qualitative assessment of image quality for myocardial delayed enhancement CT (MDE-CT). **A** Overall image quality scores of MDE-CT images reconstructed by hybrid iterative reconstruction (HIR) and deep learning image-based image reconstruction (DLIR). **B** Comparison of image quality scores assigned by two radiologists for HIR and DLIR. Each bar represents the percentage distribution across 108 patients, using a 5-point Likert scale (1 = poor to 5 = excellent)

The results of the qualitative image quality assessment are shown in Fig. 6. The overall image quality scores for DLIR images were significantly higher than those for HIR images (*p* < 0.0001). The mean scores assigned by observer 1 were 4.2 ± 0.8 for DLIR and 3.4 ± 0.8 for HIR, while observer 2 assigned scores of 3.6 ± 0.8 for DLIR and 3.2 ± 0.9 for HIR. For both observers, the proportion of patients with suitable image quality (score ≥ 3) increased when using DLIR compared to HIR (*p* < 0.03): For Observer 1, the percentage of suitable images improved from 88.9% (96/108) to 97.2% (105/108) (*p* = 0.004), and for Observer 2, from 82.4% (89/108) to 90.7% (98/108) (*p* = 0.02).

Additionally, patients were stratified into two groups based on the median body weight (60.9 kg) to evaluate whether the improvement in image quality with DLIR was consistent across different body sizes. Each weight group consisted of 54 patients (≤ 60.9 kg: *n* = 54; > 60.9 kg: *n* = 54). In both weight groups, the image quality scores for DLIR were significantly higher than those for HIR for both observers (*p* < 0.05 for all comparisons): In the lower-weight group (≤ 60.9 kg), the mean image quality scores for DLIR and HIR were 4.3 ± 0.8 and 3.5 ± 0.8 for observer 1, and 3.6 ± 0.8 and 3.3 ± 0.8 for observer 2, respectively. In the higher-weight group (> 60.9 kg), the mean scores for DLIR and HIR were 4.2 ± 0.8 and 3.4 ± 0.9 for observer 1, and 3.6 ± 0.9 and 3.2 ± 1.0 for observer 2, respectively.

### Radiation dose

In the final study cohort, the median CTDIvol and DLP for the myocardial delayed enhancement scan was 12.97 mGy (IQR, 12.58–13.64 mGy) and 152.47 mGy×cm (IQR, 129.77–167.28 mGy×cm), respectively. The median CTDIvol and DLP for the coronary CT angiography in this cohort was 12.54 mGy (IQR, 10.33–23.42 mGy) and 218.16 mGy×cm (IQR, 137.17–354.26 mGy×cm), respectively.

## Discussion

The present study demonstrated that DLIR significantly improved the image quality of MDE-CT compared with conventional HIR. Quantitative metrics, including image noise, CNR, and SNR, were all significantly improved with DLIR. Both CNR and SNR increased by approximately 27%, while image noise decreased by over 20% on average. Qualitative assessments by two experienced radiologists also confirmed these findings, showing significantly higher image quality scores and an increased proportion of diagnostically suitable images with DLIR. These results suggest that DLIR can enhance the diagnostic performance of MDE-CT by improving the visualization of delayed myocardial enhancement.

MDE-CT has emerged as a valuable alternative to LGE-CMR for detecting and characterizing myocardial fibrosis and scar, particularly in patients with contraindications to CMR such as implanted devices, claustrophobia, or patients on dialysis. Several studies have shown that MDE-CT can identify myocardial infarction and non-ischemic fibrosis with diagnostic accuracy comparable to LGE-CMR [6, 15, 16]. Furthermore, MDE-CT offers rapid image acquisition and broader availability, making it a practical option in emergency settings or in institutions without ready access to CMR. Its clinical utility has been demonstrated in assessing myocardial viability, guiding therapeutic decisions, and predicting outcomes in both ischemic and non-ischemic cardiomyopathies [15, 17–22].

However, MDE-CT is subject to several image quality–limiting factors. Myocardial delayed enhancement is typically subtle in contrast, making it highly susceptible to degradation from image noise, motion artifacts, beam-hardening, and partial volume effects. These challenges are further compounded by the fact that MDE-CT is frequently performed in the same session as coronary CT angiography. To minimize cumulative radiation exposure, MDE-CT protocols often employ low-dose acquisition settings. In addition, low-energy imaging techniques, such as low tube voltage acquisition, are frequently adopted in MDE-CT to further enhance iodine contrast [14]. While such protocols are advantageous for reducing radiation dose and enhancing iodine contrast, they also increase the risk of poor signal-to-noise ratio, potentially limiting diagnostic reliability. These inherent limitations underscore the need for advanced reconstruction techniques that can improve the image quality of MDE-CT. In this context, DLIR may offer a promising solution. By leveraging deep convolutional neural networks trained on high-quality image datasets, DLIR can suppress image noise more effectively than conventional iterative techniques while preserving fine anatomical details and image texture [23, 24]. A recent study has applied deep learning–based reconstruction to MDE-CT and demonstrated improved image quality and inter-observer reproducibility compared with conventional reconstruction methods [25]. Our study showed that DLIR significantly improved image quality metrics—reducing noise by over 20% and increasing both CNR and SNR by approximately 27%. Subjective evaluations by two radiologists confirmed these findings, with higher image quality scores and a greater proportion of diagnostically suitable images in DLIR reconstructions. The increased proportion of diagnostically suitable images observed with DLIR may help reduce inter-reader variability and thereby improve the consistency of diagnostic interpretation in clinical settings. Although the impact on downstream clinical decision-making requires further investigation, the enhanced image quality achieved with DLIR supports its value in strengthening the technical robustness of MDE-CT. These findings underscore the potential of DLIR as a meaningful component in the ongoing optimization of cardiac CT protocols.

The substantial improvement in image quality achieved by DLIR may allow for further radiation dose reduction in MDE-CT protocols. However, the extent to which DLIR can contribute to dose reduction remains uncertain based on the present study. In particular, because the current MDE-CT protocol incorporates image averaging of four separate acquisitions to improve image quality, future investigations should explore whether suitable diagnostic quality can be achieved with fewer phases when using DLIR. In addition, further studies with larger and more diverse patient populations are needed to determine optimal acquisition strategies and clarify the clinical scenarios in which DLIR-driven dose reduction would be most beneficial, especially for younger patients or those requiring repeated imaging.

Several limitations of this study should be acknowledged. First, this was a retrospective study conducted at a single institution, which may introduce selection bias and limit the generalizability of the findings. Second, although the TrueFidelity DLIR algorithm offers three selectable noise reduction strength levels (low, medium, and high), only the highest strength level was evaluated in this study. Third, ASiR-V was used with a fixed 50% blending factor, which is a commonly adopted setting in routine clinical practice. However, variations in the blending ratio could affect image quality, and different settings may yield different comparative results. In this study, we aimed to evaluate the noise reduction potential of the latest DLIR method against ASiR-V under standard clinical parameters. Fourth, this study did not include an analysis of noise texture, which may affect the subjective perception of image quality. Fifth, although our results demonstrate that DLIR improves both quantitative and qualitative image quality, the study did not evaluate diagnostic performance metrics such as sensitivity, specificity, or accuracy for detecting myocardial scar or fibrosis. Consequently, whether the observed improvements in image quality translate into enhanced diagnostic capability remains uncertain and warrants further investigation in future studies. Finally, patients without visible MDE on CT were excluded, which may have introduced selection bias and precluded assessment of false-positive findings.

In conclusion, this study demonstrates that DLIR significantly improves both quantitative and qualitative image quality in myocardial delayed enhancement CT compared to conventional HIR. By reducing image noise and enhancing the visibility of myocardial scars, DLIR may improve the diagnostic confidence. These findings support the incorporation of DLIR into routine MDE-CT protocols and highlight its potential to expand the clinical utility of cardiac CT for myocardial tissue characterization. Future studies are warranted to validate these results in larger, multi-center cohorts and to explore the impact of DLIR on diagnostic accuracy and clinical outcomes.

## Data Availability

Data will be made available on reasonable request.
